# Human Molecular Chaperone Hsp60 and Its Apical Domain Suppress Amyloid Fibril Formation of α-Synuclein

**DOI:** 10.3390/ijms21010047

**Published:** 2019-12-19

**Authors:** Hanae Yamamoto, Naoya Fukui, Mayuka Adachi, Eiichi Saiki, Anna Yamasaki, Rio Matsumura, Daichi Kuroyanagi, Kunihiro Hongo, Tomohiro Mizobata, Yasushi Kawata

**Affiliations:** 1 Department of Chemistry and Biotechnology, Graduate School of Engineering, Tottori University, Tottori 680-8552, Japan; d15t3103y@edu.tottori-u.ac.jp (H.Y.); fukui.bio@tottori-u.ac.jp (N.F.); daichi.kuroyanagi@gmail.com (D.K.); hongo@tottori-u.ac.jp (K.H.);; 2 Department of Biomedical Science, Institute of Regenerative Medicine and Biofunction, Graduate School of Medical Science, Tottori University, Koyama-Minami, Tottori 680-8552, Japan; m19m9003c@edu.tottori-u.ac.jp (M.A.); ihrg1ymya@gmail.com (A.Y.); rio.de.janeiro.0118@gmail.com (R.M.); 3 Department of Chemistry and Biotechnology, Faculty of Engineering, Tottori University, Koyama-Minami, Tottori 680-8552, Japan; eiichisaiki@s.okayama-u.ac.jp; 4 Center for Research on Green Sustainable Chemistry, Koyama-Minami, Tottori University, Tottori 680-8552, Japan

**Keywords:** molecular chaperone, human Hsp60, apical domain, α-synuclein, amyloid fibril suppression

## Abstract

Heat shock proteins play roles in assisting other proteins to fold correctly and in preventing the aggregation and accumulation of proteins in misfolded conformations. However, the process of aging significantly degrades this ability to maintain protein homeostasis. Consequently, proteins with incorrect conformations are prone to aggregate and accumulate in cells, and this aberrant aggregation of misfolded proteins may trigger various neurodegenerative diseases, such as Parkinson’s disease. Here, we investigated the possibilities of suppressing α-synuclein aggregation by using a mutant form of human chaperonin Hsp60, and a derivative of the isolated apical domain of Hsp60 (Hsp60 AD(Cys)). In vitro measurements were used to detect the effects of chaperonin on amyloid fibril formation, and interactions between Hsp60 proteins and α-synuclein were probed by quartz crystal microbalance analysis. The ability of Hsp60 AD(Cys) to suppress α-synuclein intracellular aggregation and cytotoxicity was also demonstrated. We show that Hsp60 mutant and Hsp60 AD(Cys) both effectively suppress α-synuclein amyloid fibril formation, and also demonstrate for the first time the ability of Hsp60 AD(Cys) to function as a mini-chaperone inside cells. These results highlight the possibility of using Hsp60 AD as a method of prevention and treatment of neurodegenerative diseases.

## 1. Introduction

In cells, quality control mechanisms that monitor whether proteins have attained the correct conformations exist, along with a corresponding degradation mechanism that removes proteins with incorrect conformations [1]. However, the ability to maintain these mechanisms decline during the aging process. Consequently, proteins with wrong conformations are prone to aggregate and accumulate in cells, and this aberrant aggregation of misfolded proteins contributes to various neurodegenerative diseases, such as Parkinson’s disease (PD), Alzheimer’s disease, and Polyglutamine (polyQ) diseases including Huntington’s disease [2]. The implicated proteins are different for each disease; i.e., α-synuclein for PD, amyloid β for Alzheimer’s disease, and huntingtin for Huntington’s disease. These proteins often form amyloid fibrils when they are denatured and accumulate. Amyloid fibrils and the intermediate molecular species formed during fibrillization are thought to damage cells and consequently trigger the onset of disease [3].

α-synuclein is an intrinsically disordered protein consisting of an amphiphilic N-terminus, a hydrophobic middle region that corresponds to the nucleus of amyloid fibrils, and acidic C-terminal regions [4]. During fibrillation of this protein, three characteristic phases are commonly observed: (i) the initial lag phase, in which unfolded monomers aggregate and associate to form nuclei, (ii) the growth phase, in which monomers associate with the nucleus and rapid nucleus-dependent fibril extension occurs, and (iii) the final plateau phase, in which the fibril extension is saturated and fibrils are matured [5,6,7]. Cellular inclusions called Lewy bodies has been found in midbrain substantia nigra neurons of PD patients, the main component of which is known to be amyloid fibrils of α-synuclein [8]. Since various molecular chaperones such as Hsp27, Hsp60, and Hsp70 have been reported to colocalize with α-synuclein in Lewy bodies [9], chaperones may potentially play a role in PD progression [10].

The heat shock response is the cell response that occurs when an organism is exposed to a temperature higher than normal growth temperature, and a group of proteins that are induced under such conditions are collectively called heat shock proteins (Hsps). Hsps, which act to protect cells from stress damage, function as molecular chaperones that help correct protein folding [11]. Hsps, for example, small heat shock proteins (sHsps), Hsp60, Hsp70, Hsp90, and Hsp100 are named according to their constituent polypeptide molecular weights. The role and therapeutic potential of sHsps in preventing the toxicity associated with intracellular α-synuclein aggregation has been reported [12]. Hsp70 and Hsp90 based chaperone-like peptides (“mini-chaperones”) have also been used in cancer immunotherapy and vaccine development [13].

Hsp60 is one of the most well-studied of the molecular chaperones. Hsp60 chaperones are divided into two groups in terms of structure, group I and group II. Group I Hsp60s are present in eubacteria, mitochondria, chloroplasts, etc., and generally function as a chaperone in cooperation with a co-chaperonin, Hsp10. Group II Hsp60s in contrast are present in the cytoplasm of archaebacteria and eukaryotes, and they possess a unique structure in which a co-chaperonin-like structure is incorporated. CCT is a group II Hsp60 molecular chaperone that localizes to the cytoplasm. Sot et al. [14] reported that CCT showed a suppression effect on α-synuclein aggregation. Moreover, a subset of CCT subunits also showed the suppression effect of huntingtin aggregation, suggesting its therapeutic possibilities [15]. The mitochondrial Hsp60 is a quality control protein that functions as two heptameric rings stacked back to back and maintains mitochondrial function. Hsp60 plays a role in assisting the folding of other proteins with Hsp10 in an ATP-dependent manner in the mitochondrial matrix [16,17]. Hsp60 has also been reported to associate with the PD-related proteins: α-synuclein, DJ-1, PINK1, and Parkin [18,19,20]. It has been reported that Hsp60 induced behavioral improvements in PD model rats [21]. Furthermore, a systems biology analysis of PD model cells revealed that Hsp60 was the central hub in the protein–protein interaction network [22]. Recently, it has also been reported that Hsp60 exhibits a suppressive effect on Aβ_1–40_ amyloid fibril formation in vitro [23]. However, an analogous effect on the part of Hsp60 to suppress aggregation or amyloid fibril formation of α-synuclein has not yet been demonstrated in detail.

Previous studies have shown that the apical domain of the group I Hsp60 GroEL has a strong influence on α-synuclein amyloid fibrillation. We found that the apical domain (AD) open-mimic mutant GroEL, of which the glycine(G)192 at hinge II was mutated to tryptophan(W), has a powerful suppressive effect on amyloid fibril formation [24]. In addition, it has been shown that the isolated GroEL AD alone suppresses amyloid fibrillization of proteins such as α-synuclein and Aβ_1–42_, and functions as a mini-chaperone [25]. Being derived from a bacterial source, however, GroEL shows limited potential in application to therapeutic research for mammals due to the differences in origin species. Therefore, in this study, we studied the suppression effect of human Hsp60 on α-synuclein amyloid fibrillization. We show that the orientation of the apical domain in Hsp60 is important in suppressing α-synuclein amyloid fibrillation. Furthermore, we demonstrate that isolated AD of Hsp60 (Hsp60 AD) itself is able to effectively suppress α-synuclein aggregation and cytotoxicity in cultured cells. This finding suggests that Hsp60 AD might be a potential therapeutic agent for neurodegenerative diseases, such as PD.

## 2. Results and Discussion

Chaperones are essential for maintaining protein homeostasis, and the ability to suppress aggregation is in line with the general functional behavior of molecular chaperones. Various reports have outlined the ability of chaperones, except Hsp60, to suppress the accumulation and aggregation of α-synuclein [14,26,27,28,29]. Here we show that the human chaperonin Hsp60 and its apical domain are able to interact with α-synuclein monomers and suppress its fibrillization in vitro for the first time. We also investigated the function of Hsp60 AD as a mini-chaperone inside cells.

### 2.1. Open-Mimic Mutant Hsp60 G190W (GW) Is a Potent Suppressor of Amyloid Fibril Formation

**Figure 1:**
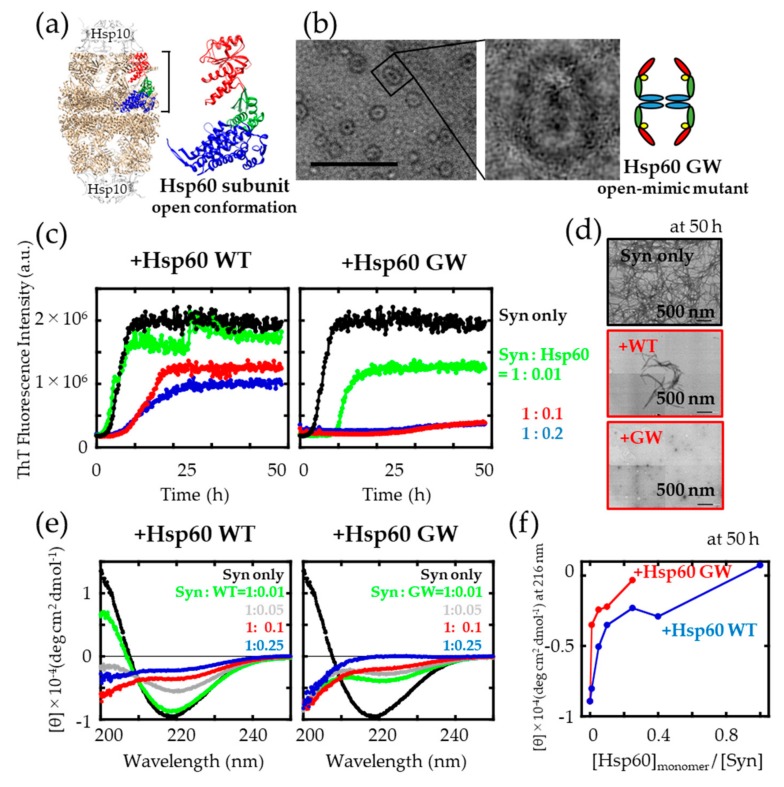
Influence of Hsp60 wild type (WT) and GW on α-synuclein aggregation. (**a**) The open conformation of Hsp60 (PDB ID: 4PJ1), using UCSF Chimera [32]. Left: overall side profile structure of PDB ID: 4PJ1. Right: Hsp60 subunit in the open conformation. Apical domain (red), intermediate domain (green), and equatorial domain (blue). (**b**) TEM observation and simplified side view of Hsp60 GW. Scale bar = 50 nm. (**c**) Time course of Thioflavin T (ThT) fluorescence changes upon incubation of α-synuclein in the absence (black) and presence of a 0.01 (green), 0.1 (red) and 0.2 (blue) fold concentration (monomer: monomer) of Hsp60. (**d**) TEM images of α-synuclein samples shaken in either the absence or presence of a 0.1 monomer-molar ratio of Hsp60 (scale bar: 500 nm). (**e**) Circular dichroism (CD) spectra of α-synuclein samples recorded at 25 °C after agitation. The raw data were corrected for contributions of native Hsp60 by subtraction. (**f**) Hsp60 concentration-dependent changes in the CD absorbance signal at 216 nm.

Our previous studies showed that the GroEL G192W mutant “locks” the GroEL oligomer in an open conformation through introduction of tryptophan at hinge II site pushes AD upward. This enhances the affinity of GroEL G192W toward fibrillogenic polypeptides [24,30]. We intended to apply this method to human Hsp60 (Figure 1a) in this study.

The human Hsp60 GW, corresponding to G192W in GroEL [31], was utilized to investigate the effects of changes in the ring conformation on the ability to prevent protein fibrillation. TEM observation revealed that the side view of Hsp60 GW resembled a football shape, similar to GroEL G192W, that suggested a tendency of this protein to assume an open conformation (Figure 1b). We compared the dose-dependent suppression effects of Hsp60 wild type (WT) and Hsp60 GW on the amyloid fibril forming reaction of α-synuclein by monitoring the fluorescence signal of Thioflavin T (ThT) dye (Figure 1c). ThT emits a very strong fluorescence when bound to β-sheets in amyloid fibrils. α-synuclein was allowed to form fibrils at 37 °C under continuous shaking in the absence or presence of various molar ratios of Hsp60 (based on monomer concentration). In the presence of a 1:10 molar ratio of Hsp60 GW to α-synuclein, the final ThT fluorescence intensity was strongly suppressed; however, this concentration of Hsp60 WT was not sufficient to suppress the fluorescence signal completely. Figure 1d shows TEM images of α-synuclein samples prepared in the presence of Hsp60. In samples of α-synuclein fibrils formed in the absence of chaperonin, typical amyloid fibrils were observable throughout the sample mesh grid. In contrast, sample grids of α-synuclein fibrils formed in the presence of 0.1-fold Hsp60 WT, contained observable fibrils in only portions of the grid. No fibrils were observed in the sample of α-synuclein with 0.1 monomer-molar ratio of Hsp60 GW.

Next, we measured circular dichroism (CD) to investigate the effect of Hsp60 on the secondary structure of α-synuclein. Figure 1e indicates CD spectra of α-synuclein after agitation for 50 h in the presence and absence of various concentrations of Hsp60. We note here that the raw CD spectra of samples were corrected for the presence of Hsp60 by subtraction of the spectra of Hsp60 measured under identical conditions. α-synuclein fibril samples formed in the absence of Hsp60 showed CD spectra with a negative maximum at 216–218 nm, which indicated a high fraction of β-sheet structure. In the presence of different concentrations of Hsp60, the CD spectra of α-synuclein samples changed in a dose-dependent manner. As exemplified in Figure 1f for the CD absorbance observed at 216 nm, the addition of Hsp60 GW decreases the β-sheet structure content of α-synuclein dramatically in a dose dependent manner.

### 2.2. Hsp60 GW Shows an Increased Degree of Surface Hydrophobicity Due to Open-Mimic AD Conformation

**Figure 2:**
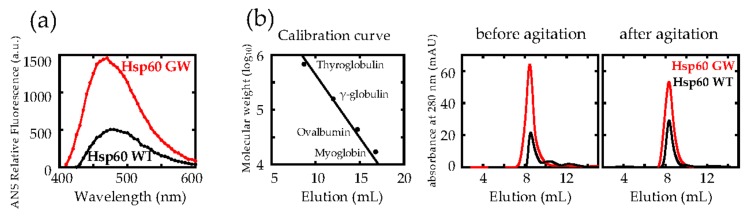
Comparing the structural characteristics of wild type (WT) and G190W Hsp60. (**a**) Estimation of protein surface hydrophobicity by ANS binding. (**b**) Evaluation of Hsp60 quaternary structure using gel-filtration chromatography. Hsp60 samples were applied to a Superdex 200 Increase 10/300 GL column and the relative molecular size was estimated from a curve calibrated with the following molecular weight standards: Thyroglobulin (bovine) 670,000, γ-globulin (bovine) 158,000, Ovalbumin (chicken) 44,000, and Myoglobin (horse) 17,000.

In order to determine whether the G190W mutation introduced into Hsp60 resulted in a change in surface hydrophobicity of this chaperonin, we next used the fluorescence probe ANS which was used to detect subtle hydrophobicity changes [33,34]. We observed that the ANS fluorescence intensity for Hsp60 GW was greater compared to that of WT (Figure 2a). This result reflected a high relative surface hydrophobicity in Hsp60 GW compared to wild type.

As shown in Figure 2b, estimation of the relative molecular weights of Hsp60 WT and GW using gel-filtration chromatography (Superdex 200 Increase 10/300 GL column) revealed that the quaternary structure of Hsp60 was similar, indicating that the oligomeric structure of Hsp60 was largely unaffected by the mutation and that the increase in hydrophobicity could not be attributed to subunit dissociation. Furthermore, we examined whether the agitation utilized in Figure 1c affected the Hsp60 quaternary structures. Since there were almost no differences between the elution volumes before and after agitation, it was likely that neither mutation nor agitation affected the quaternary structure. The equation for the calibration curve was determined from four marker proteins; *y* = 7.9341−0.22968 *x*, *R*^2^ = 0.9914. Each Hsp60 revealed a single peak with elution volume of 8.34–8.57 mL (corresponding molecular weight: 1,044,000–924,000). We conclude that both Hsp60 WT and GW are stable tetradecamers, supporting a previous report [35]. Taken together with the results of GroEL G192W [24,30], we concluded that Hsp60 G190W had the open-mimic conformation of AD, similar to GroEL G192W, with hydrophobic characteristics that probably enhanced the amyloid fibrillation suppression effect.

### 2.3. Hsp60 WT and GW Interact with α-Synuclein Monomers

**Figure 3:**
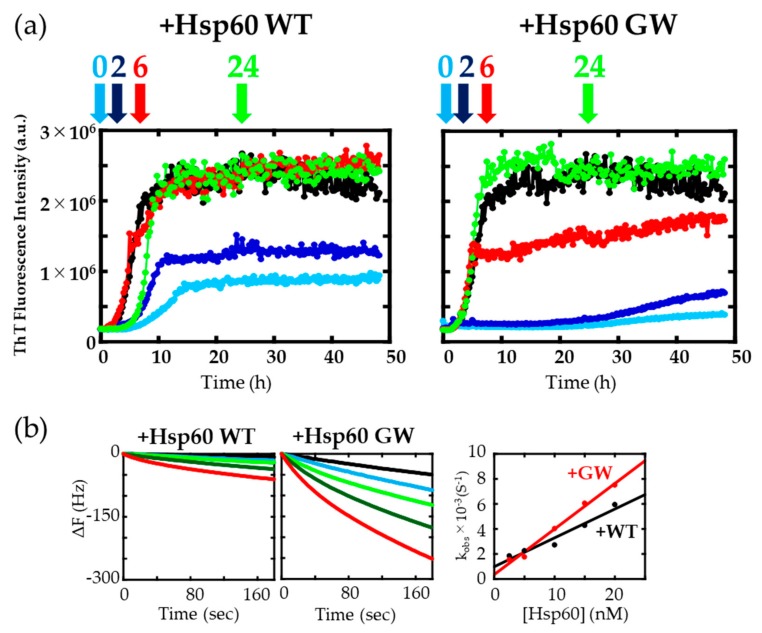
(**a**) Effects of delayed chaperone addition on the aggregation process of α-synuclein. Hsp60 (0.1 monomer-molar ratio) was added at 0 h (light blue), 2 h (dark blue), 6 h (red), and 24 h (green) after initiation of fibrillization. The black trace indicates α-synuclein without Hsp60. (**b**) Binding of Hsp60 to immobilized α-synuclein monomers. The time course of changes in frequency of the His-α-synuclein-immobilized quartz crystal microbalance (QCM) sensor upon addition of 2.5 nM (black), 5 nM (light blue), 10 nM (light green), 15 nM (dark green), and 20 nM (red) of Hsp60. Right: k_obs_ versus concentration of Hsp60.

To understand the suppression mechanism, we next investigated the effects of adding Hsp60 WT and GW at various stages of α-synuclein aggregation. Hsp60 WT and GW were added at the beginning (0 h) or the middle (2, 6, 24 h) of the fibrillation reaction, while monitored by ThT fluorescence (Figure 3a). Adding Hsp60 WT and GW at an early stage of the reaction succeeded in suppressing α-synuclein aggregation and was especially effective when added prior to starting the reaction (0 h). When Hsp60 WT and GW were added at 2 h (and 6 h for GW) agitation, the ThT fluorescence intensities were halted, suggesting that Hsp60 WT and GW bind α-synuclein monomer or fibril intermediate. In contrast, addition of Hsp60 WT and GW to mature amyloid fibrils (24 h) did not result in a change in fluorescence, which indicated that Hsp60 WT and GW could not disassemble amyloid fibrils to monomers once formed. Contrasting with our results, Gong et al. [36] reported that delayed addition of 1,4-benzoquinone and 1,4-naphthoquinone showed a partial disaggregation effects towards insulin amyloid fibrils.

To detect interactions between Hsp60 WT/GW and α-synuclein, initially we used immunoprecipitation and Western blot analysis (Figure S1). As shown in Figure S1, protein bands reflecting association between these two proteins were detected under all conditions, indicating that both Hsp60 WT and Hsp60 GW bound to α-synuclein in a specific manner.

To directly evaluate the binding affinities between Hsp60 WT/GW and α-synuclein monomer, we employed quartz crystal microbalance (QCM) analysis, a sensitive technique that determines binding affinities through direct measurement of a change in bound mass to a quartz crystal plate vibrating at a constant frequency. Since the weight of molecules that bind is proportional to the amount of frequency change in the quartz microbalance, the number of molecules bound may be determined. Figure 3b (left and middle) shows the sensorgrams of interactions between α-synuclein monomers and Hsp60 WT/GW at various Hsp60 concentrations. Upon addition of higher concentrations of Hsp60 WT/GW to the QCM sensor containing immobilized α-synuclein, an increase in ΔF was observed. The sensorgrams obtained from Hsp60 GW showed a larger ΔF value compared to WT, which suggested that GW has a stronger binding affinity to α-synuclein. The reciprocal of the binding relaxation time (τ), k_obs_, for each binding process against α-synuclein was plotted against the concentration of Hsp60 and was found to correlate linearly (Figure 3b, right). The correlation coefficients of linearities were *R*^2^ = 0.951(WT) and R^2^ = 0.989(GW). The obtained k_on_, k_off_, and equilibrium dissociation constant K_d_ (= k_off_/k_on_) values are summarized in Table 1. Estimated K_d_ values of Hsp60 WT and GW toward immobilized α-synuclein were 0.963 nM for GW and 4.29 nM for WT. This is a significant difference when we consider that the higher surface hydrophobicity of GW (as reflected in the results in Figure 2a) would contribute to the stability of the interaction, resulting in a higher k_on_ value and a lower k_off_ value when compared to WT. In separate experiments (Figure S2), we confirmed that K_d_ values measured by QCM correlated very well with values derived from fluorescence cross-correlation spectroscopy experiments [16], which suggests that these estimates regarding the affinity between Hsp60 and α-synuclein are a reasonable approximation.

### 2.4. Stabilization of Isolated Hsp60 AD

The apical domain of Hsp60 family is known to be the domain that is responsible for the interaction with target proteins [37,38,39], and many studies have reported the ability of this domain to act as mini-chaperones in isolated form [15,40,41]. The paper by Tam et al. [41] indicated that the apical domain of CCT1 suppressed polyQ aggregation. Ojha et al. [25] found that the isolated GroEL AD suppressed α-synuclein and Aβ_42_ amyloid formation.

**Figure 4:**
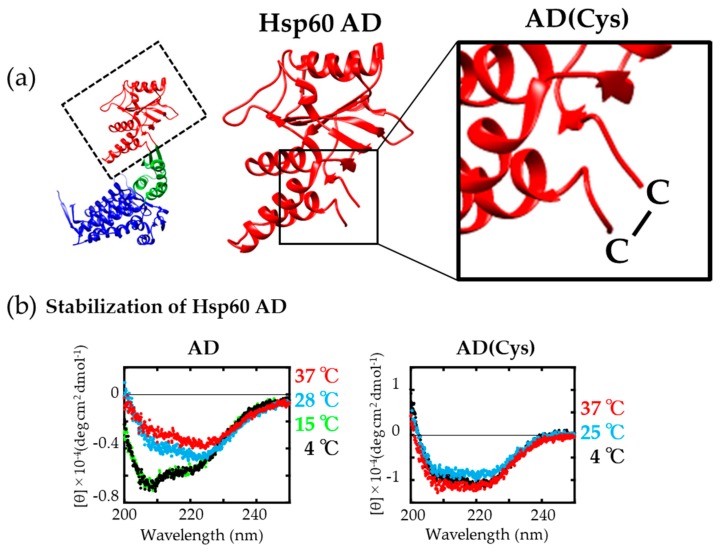
(**a**) Structure of Hsp60 monomer (left) and the apical domain (middle) (PDB ID: 4PJ1). (**b**) CD spectra of Hsp60 apical domain (AD) (left) and AD(Cys) (right). Each Hsp60 AD was incubated overnight at the indicated temperatures prior to measurement.

Since Figure 1 and Figure 3 indicated that AD was very important for chaperone function, we prepared and purified the isolated AD as soluble protein to explore the possibilities of utilizing AD as a mini-chaperone. However, we found that the secondary structure of AD was relatively unstable and susceptible to denaturation at higher temperature. As shown in Figure 4b left, the CD spectra of Hsp60 AD showed strong negative peaks at 205–210 nm and weak negative peaks at 220–225 nm at 4 and 15 °C. In contrast, the negative peaks at 205–210 nm gradually disappeared at higher temperatures (28 and 37 °C). Additionally, the solubility of lyophilized AD preparations was very poor in Tris buffer, which suggested that the AD was too unstable to withstand the lyophilization process. In order to overcome this shortcoming of the isolated AD, we introduced cysteine residues into the N- and C-termini of the AD using mutagenesis [42] and allowed these cysteines to form a disulfide bond (Hsp60 AD(Cys)) once purified. Disulfide bond formation occurred spontaneously, because the distance between the two engineered cysteine residues was very close in the AD conformation (Figure 4a right, AD(Cys)). We confirmed this disulfide bonding by using 5,5′-dithiobis-(2-nitrobenzoic acid) spectrophotometric quantitation analysis [43]. As shown in Figure 4b right, no significant change in CD spectrum was observed at higher temperatures for AD(Cys). We noticed that the CD spectra of AD at 4 °C was different from AD(Cys) as shown in Figure 4b. From secondary structure component analysis using the Beta Structure Selection website [44] and a simulation of the CD spectrum [45], it was suggested that the structure of AD seemed to be partially unfolded even at 4 °C (Figure S3). The solubility of this engineered protein was also increased, indicating that introducing a disulfide bond served to stabilize Hsp60 AD. The AD(Cys) was also found to be a monomer from the result of gel-filtration chromatography analysis.

### 2.5. Hsp60 AD Functions as a Mini-Chaperone

**Figure 5:**
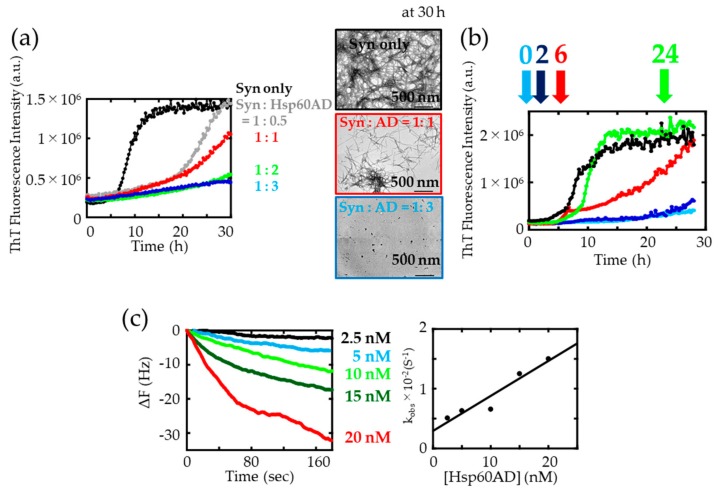
(**a**) ThT assay for the kinetics of α-synuclein fibrillation with various amounts of AD(Cys). TEM images of α-synuclein fibrils in the absence (black) and in the presence of AD(Cys) (red: one-fold and blue: three-fold) (scale bar: 500 nm). (**b**) Suppression effect analysis of delayed AD(Cys) addition on amyloid formation process. Three-fold AD(Cys) was added at 0 h (light blue), 2 h (dark blue), 6 h (red), and 24 h (green) on the fibrilization process monitored by ThT. The black trace indicates α-synuclein without AD(Cys). (**c**) Interaction analysis between α-synuclein monomer and AD(Cys) using the QCM method. The correlation coefficient of linearity of the dependence of k_obs_ on [AD(Cys)] was *R*^2^ = 0.917.

We next evaluated the suppression effects of AD(Cys) on α-synuclein amyloid fibril formation. The ThT assay and TEM measurements confirmed that AD(Cys) suppressed the formation of α-synuclein fibrils in a dose-dependent manner (Figure 5a), and addition of three-fold AD(Cys) suppressed α-synuclein amyloid formation completely. In delayed addition experiments (Figure 5b), AD(Cys) showed a strong suppression effect at the early stages of fibril formation (0 and 2 h). Moreover, interactions between AD(Cys) and α-synuclein were examined by immunoprecipitation (Figure S4) and QCM analysis (Figure 5c and Table 2). From these results, it was confirmed that a strong interaction between AD(Cys) and α-synuclein monomer was present (K_d_ = 5.04 nM). Taken together, we suggest that AD(Cys) interacts with α-synuclein monomer and suppresses α-synuclein fibrillization. These results demonstrated the activity of AD(Cys) as a mini-chaperone.

Table 1 and Table 2 show that AD(Cys) displayed greatly enhanced binding and dissociation rates compared to WT and GW. The isolated AD(Cys) exposed the hydrophobic surface region located inside the ring into the solution, so the rate of binding (k_on_) to α-synuclein was considered to be the largest. In contrast, the large dissociation rate (k_off_) implied the weakness of retaining α-synuclein monomers. Hsp60 was most likely enhancing its ability to remain bound to α-synuclein monomers by forming a ring-like oligomer. We believe that this is the reason why we required larger amounts of AD(Cys) to suppress amyloid fibril formation, compared to WT and GW (compare Figure 1c and Figure 5a, amount of added chaperone relative to Syn).

### 2.6. Hsp60 AD Suppresses α-Synuclein Aggregation and Cytotoxicity

**Figure 6:**
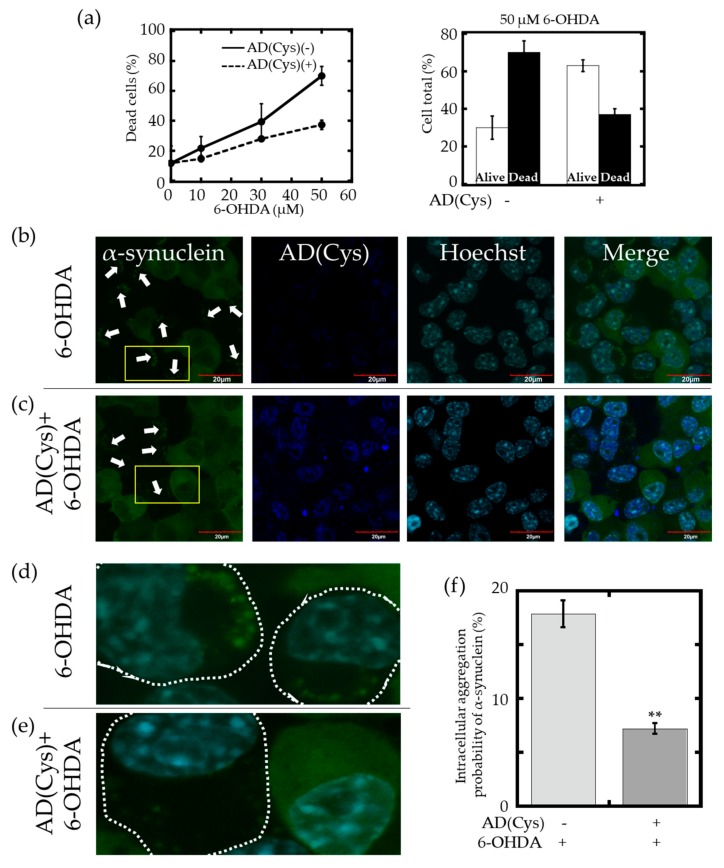
(**a**) Left: cell death rate. Data are expressed as a percentage of Ethidium Homodimer 1 (EthD-1)-positive cells. Right: the percentage of alive cells and dead cells under the oxidative stress condition of 50 μM 6-OHDA. The error bars represent ± SEM. (**b**,**c**) Immunofluorescence staining of AD(Cys) in green fluorescent protein-tagged human α-synuclein (GFP-Syn) expressing Neuro2a cells under 10 μM 6-OHDA-induced oxidative stress. The boxed regions in the images are magnified in (**d**,**e**), respectively. The white arrows indicate GFP-Syn aggregation in cells. Scale bars: 20 μm. (**d**,**e**) Enlargements of the merged GFP-Syn and Hoechst images of the yellow-boxed regions in (**b**,**c**), respectively. Cells with GFP-Syn aggregation are indicated by dotted lines. (**f**) Quantitative analysis of α-synuclein aggregation. The error bars represent ± SEM., Welch’s *t*-test, ** *p* < 0.01.

Finally, we investigated whether AD(Cys) could suppress the intracellular aggregation of α-synuclein and its accompanying cytotoxicity. A stable mouse neuroblastoma cell line (Neuro2a) expressing green fluorescent protein-tagged human α-synuclein (GFP-Syn) was established. GFP-Syn expressing Neuro2a cells formed intracellular punctate aggregates (foci) upon application of oxidative stress, induced by 6-OHDA [46]. This GFP-Syn foci served as a visual representation of the intracellular aggregation of α-synuclein which potentially leads to amyloid fibril formation. Oxidative stress, induced by 10, 30, or 50 μM 6-OHDA was applied to both AD(Cys) protein-introduced and non-introduced cells, and cells were then stained with Ethidium Homodimer 1 (EthD-1) to determine the cell death rate by Tali™ Image-Based Cytometer (Figure S5). Figure 6a (left) shows the cell death rate at various concentrations of 6-OHDA, indicating increased cell mortality depending on the concentration of 6-OHDA. In the AD(Cys)-introduced cells, a lower cell death rate was observed compared with that of AD(Cys)-deficient cells at all concentrations of 6-OHDA tested. In particular, a significant difference was obtained under the oxidative stress condition of 50 μM 6-OHDA (Figure 6a, right).

Next, in order to investigate the cause of the lower cell death rate, a confocal laser microscope observation was performed (Figure 6b–e). After 10 μM 6-OHDA was added to both the AD(Cys)-protein introduced cells and the non-introduced cells, the formation of foci after a 24-h incubation was observed. We could confirm the successful introduction of AD(Cys) into Neuro2a cells, as shown in Figure S6. As shown in Figure 6b,d, numerous foci of GFP-Syn aggregates were observed for AD(Cys)-deficient cells. Interestingly, as shown in Figure 6c,e, the number of GFP-Syn foci seemed to be smaller in cells that retain AD(Cys). The ratio of cells containing GFP-Syn foci were analyzed manually from confocal images, by counting both the total cell number (T) and the number of cells with aggregation (A) and deriving the aggregation probability (100A/T), as shown in Figure 6f. The experiments were repeated three times, and over 200 cells were quantified for each experiment. Foci were observed in an average of 18% of the cells that did not retain AD(Cys), likely due to oxidative stress. In AD(Cys)-introduced cells, this ratio of foci-positive cells decreased to an average of 7%, indicating that a significant decrease of intracellular α-synuclein aggregation had occurred. Since the introduction of AD(Cys) did not affect the expression of neither GFP-Syn (Figure S7), endogenous Hsp60 nor Hsp70, we may deduce that AD(Cys) acted to suppress GFP-Syn aggregation in these cells. These results demonstrated that AD(Cys) could function effectively as a mini-chaperone in cells as well.

We noticed that a fraction of introduced AD(Cys) also localized to the nucleus in Figure 6c and Figure S6. To understand the reason for this dual localization, the subcellular localization probability of AD(Cys) was predicted by using a prediction algorithm [47]. The results showed a high probability of nuclear localization (25%) that was only slightly lower than the localization propensity to the cytoplasm (27%). Thus, the localization of AD(Cys) in the nucleus may be attributed to its amino acid sequence.

It has been reported that Hsps introduced by using cell-penetrating peptides, protected cells from various cytotoxic treatments [48,49,50,51]. Sontag et al. indicated that the isolated apical domain of cytosolic chaperonin CCT1 in type of group II could enter cells and reduced huntingtin aggregation [15]. Their results suggested that mini-chaperone could be developed as drugs for amyloid related diseases. In the present study, we also demonstrated that AD(Cys) of group I Hsp60 interacts with α-synuclein to suppress aggregation, i.e., works as a mini-chaperone efficiently even in cells that are often described as “multi-macromolecular crowding” for the very first time, although stability of the AD(Cys) have not been clarified in cells [52].

**Figure 7:**
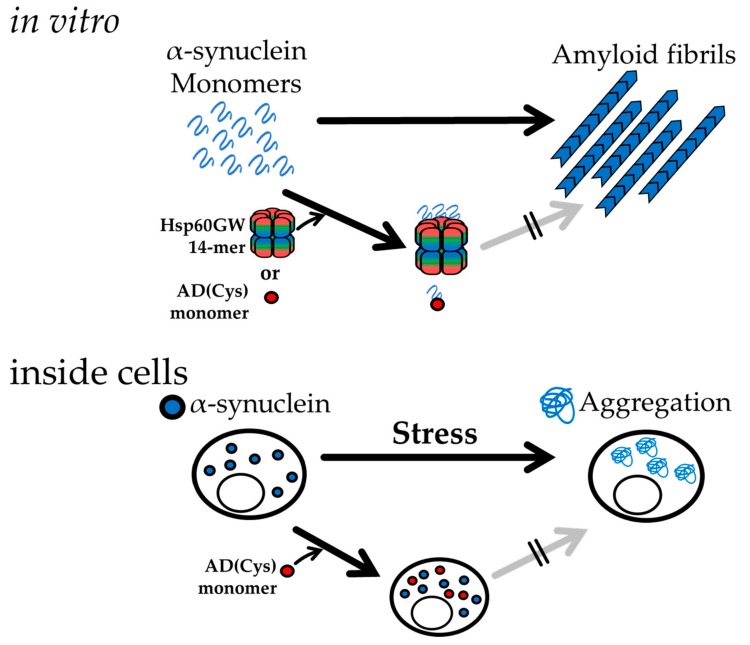
A mechanism model that Hsp60 or AD functions on suppression of α-synuclein amyloid fibril or aggregation formation.

Taken together with the in vitro experimental results, we could summarize the suppression abilities of Hsp60 GW and AD(Cys) as shown in Figure 7. Human Hsp60 GW, in which the apical domain is fixed in an open conformation, is able to interact with α-synuclein monomer easily, and AD(Cys) suppressed amyloid fibril formation of α-synuclein in vitro. This was performed by binding of α-synuclein monomer to Hsp60 GW and AD(Cys). We could not determine whether Hsp60 GW and AD(Cys) preferentially bound to monomeric or oligomeric species of α-synuclein in our experiments, but the possibility of preferential binding to either one of these two species cannot be excluded. Although the detailed suppression mechanism of α-synuclein aggregation in the cells is not clear, we determined that AD(Cys) is a functional mini-chaperone even inside cells. This result holds potential for the eventual development of a medical treatment for PD.

## 3. Materials and Methods

### 3.1. Materials

Unless otherwise stated, all chemical reagents were obtained from commercial suppliers and used without any further purification.

### 3.2. Protein Expression and Purification

Genes encoding codon-modified human Hsp60 WT were amplified by polymerase chain reaction (PCR). The pair of primers was 5′-GTA CCC ATA TGG CCA AAG ATG TTA AAT TTG-3′ (forward) and 5′-TGG CCA TAT GGG TAC CGC GGA CAA GAC ACG-3′ (reverse). The PCR product and pET23a(+) vector (Merck, Darmstadt, Germany) were ligated after restriction enzyme (Nde I and Sac I (Takara Bio, Shiga, Japan)) digestions. The pET23a(+)-hsp60 WT plasmid was transformed into *Escherichia coli* (*E. coli*) BLR(DE3). Cells were cultured overnight at 37 °C in LB agar plates containing 50 μg/mL ampicillin. The bacterial cells were grown in LB medium containing 50 μg/mL ampicillin at 37 °C to an OD_600_ of 0.6 and then induced by addition of 1 mM isopropyl β-D-1-thiogalactopyranoside (IPTG). Protein expression was continued overnight at 37 °C. The cells were harvested by centrifugation and the cell pellets were suspended in lysis buffer containing 50 mM Tris-HCl (pH 7.8 at 25 °C), 2 mM DTT, 2 mM EDTA, 0.1 mM PMSF (Phenylmethylsulfonyl fluoride), and final concentration 1 mg/mL of lysozyme. The suspension was disrupted using sonication and centrifuged. Streptomycin sulfate was added to the supernatant and centrifuged. Hsp60 WT protein precipitated using ammonium sulphate precipitation (55% saturation) and centrifuged. The pellet was suspended in buffer (50 mM Tris-HCl (pH 7.8 at 25 °C), 2 mM DTT, 2 mM EDTA). This concentrated protein solution was loaded onto a size exclusion chromatography column (Superdex 200 Increase 10/300 GL, GE Healthcare Life Sciences, Marlborough, MA, USA). Flow rate was 0.75 mL/min. Fractions containing Hsp60 WT was analyzed by SDS-PAGE, collected and loaded onto an anion-exchange chromatography column (Resource Q 6 mL, GE Healthcare Life Sciences, Marlborough, MA, USA). Flow rate was 2 mL/min, NaCl gradient was 0–0.6 M (10 column volumes). Fractions containing Hsp60 WT were desalted by dialysis and stored at 4 °C.

Genes encoding the human Hsp60 GW mutant were obtained using a PCR-based site-directed mutagenesis method to make a specific change from a small amino acid residue (glycine) to a bulky tryptophan at hinge II of hsp60 WT. The two primers were 5′-ATC GAA TGG ATG AAA TTC GAT CGC GGT TA-3′ (forward) and 5′-TTT CAT CCA TTC GAT AAT TTC CAG TTC ATC-3′ (reverse). The PCR product was inserted into the pET24a(+) expression plasmid (Merck, Darmstadt, Germany) using Nde I and Sac I restriction sites. The pET24a(+)-hsp60 GW plasmid was transformed into *E. coli* JM109(DE3). Cells were cultured overnight at 37 °C in LB agar plates containing 30 μg/mL kanamycin. Bacteria were grown at 25 °C overnight in 2 × TY medium containing 30 μg/mL kanamycin. The cells were harvested by centrifugation. The cell pellets were sonicated and centrifuged as Hsp60 WT. The purification of Hsp60 GW was followed by ammonium sulphate precipitation, size-exclusion chromatography, anion-exchange chromatography (0–0.45 M NaCl gradient (12 column volumes)) and dialysis.

Genes encoding Hsp60 AD were amplified by PCR. The primers were 5′-ACC ATG GGC ATG AAA TTC GAT CGC GGT TAT A-3′ (forward) and 5′-GTG GTG ATC ACT CAG TTT TGC CAG ACG TTC A-3′ (reverse). Genes encoding pET28a(+) were amplified using following primers by PCR: 5′-CTG AGT GAT CAC CAC CAC CAC CAC CAC TGA GAT C-3′ (forward) and 5′-TTT CAT GCC CAT GGT ATA TCT CCT TCT TAA AGT T-3′ (reverse). The hsp60 AD genes were inserted into pET28a(+) vector using the In-Fusion HD Cloning Kit (Takara Bio, Shiga, Japan). Furthermore, cysteine residues were introduced into both the N- and C- termini of hsp60 AD genes by PCR to produce a gene coding for Hsp60AD(Cys). The primers for N-terminal were 5′-ACC ATG TGC GGC ATG AAA TTC GAT CGC GGT T-3′ (forward) and 5′-CAT GCC GCA CAT GGT ATA TCT CCT TCT TAA AGT T-3′ (reverse). The primers for C-terminal were 5′-AGT GAT TGC CAC CAC CAC CAC CAC CAC TGA-3′ (forward) and 5′-GTG GTG GCA ATC ACT CAG TTT TGC CAG ACG-3′ (reverse). A single colony from BLR(DE3)/pET28a(+)-hsp60AD LB agar plate (30 μg/mL kanamycin) was cultured in LB medium containing 30 μg/mL kanamycin at 37 °C. Hsp60 AD expression was induced with IPTG once the OD_600_ reached 0.6, and the cells were grown overnight at 37 °C. The bacterial suspension was spun and collected. The cell pellets were suspended in lysis buffer containing 50 mM Tris-HCl (pH 7.8 at 25 °C), 2 mM EDTA, 0.1 mM PMSF, and 1 mg/mL of lysozyme. The suspension was disrupted using sonication and centrifuged. Streptomycin sulfate (final 2.5%) was added to the supernatant and centrifuged. The clear supernatant of cell lysate was desalted by dialysis. The solution was loaded onto a Ni–Sepharose column (HisTrap HP, GE Healthcare Life Sciences, Marlborough, MA, USA). The bound proteins were eluted with 0 to 0.5 M imidazole gradient (13 column volumes) and eluted fractions were analyzed by SDS-PAGE. The fractions rich-containing Hsp60 AD were removed imidazole by dialysis. The sample was loaded onto anion-exchange column (Resource Q 6 mL, 0–0.3 M NaCl gradient (13 column volumes)). Fractions containing Hsp60 AD were desalted by dialysis, then lyophilized and stored at 4 °C.

α-synuclein and Histidine-Tagged (His-) α-synuclein were expressed in *E. coli* and purified as described previously [53].

### 3.3. Thioflavin-T (ThT) Fluorescence Assays

α-synuclein samples (0.5 mg/mL (Figure 5a) or 1 mg/mL (other figures)) were incubated with or without Hsp60 variants at 37 °C in 25 mM Tris-HCl buffer (pH 7.4 at 37 °C) containing 150 mM NaCl and 20 μM ThT. Samples were added into wells with polytetrafluoroethylene beads on 96-well microplates (polystyrene plate, black with transparent bottom; Greiner Bio-One, Kremsmünster, Austria) in sextuplicate (Figure 5a) or triplicate (other figures) (150 μL/well). Each plate was incubated under orbital shaking at 37 °C and measured at 15-min intervals from the bottom (ARVO X4, PerkinElmer, Waltham, MA, USA). The fluorescence was excited at 450 nm and detected at 486 nm.

### 3.4. Transmission Electron Microscopy (TEM)

TEM images were obtained using a JEM-1400Plus transmission electron microscope (JEOL, Tokyo, Japan) at 80 kV. The carbon-coated 400-mesh copper grids (Nisshin EM, Tokyo, Japan) was hydrophilized with Ion Sputtering device E1010 (Hitachi, Tokyo, Japan). Hsp60 GW was diluted with buffer (25 mM Tris, 150 mM NaCl, 20 mM KCl, 10 mM Mg(CH_3_COO)_2_) to a concentration of 0.3 mg/mL, and deposited onto the grids for 2 min. Then, a final concentration of 2% glutaraldehyde was added, incubated for 2 min, and the grids were washed twice with the buffer. After negative staining with 2% uranyl acetate, the grids were washed twice. α-synuclein samples in the presence or absence of Hsp60 variants were applied to carbon-coated 400-mesh copper grids (Nisshin EM, Tokyo, Japan), washed once with ultrapure water. After negative staining with EM Stainer (Nisshin EM, Tokyo, Japan), the grids were washed once with ultrapure water. Grids were incubated for more than one night at room temperature to dry completely before TEM analysis.

### 3.5. Circular Dichroism (CD)

α-synuclein samples in the presence or absence of Hsp60 GW (or WT) were diluted to a final concentration of 0.2 mg/mL in 25 mM Tris buffer (pH 7.7 at 25 °C). For α-synuclein samples in the presence of Hsp60 GW (or WT), spectra of Hsp60 GW (or WT) were subtracted to obtain the net spectra of α-synuclein. Stability of Hsp60 AD was analyzed using 0.05–0.1 mg/mL proteins with buffer containing 25 mM Tris buffer (pH 7.7 at 25 °C) and 150 mM NaCl. Hsp60 AD samples were incubated overnight at 4–37 °C. The samples were measured on a J-820 CD spectrometer (JASCO, Tokyo, Japan) using a 0.1 cm path length synthetic quartz cuvette (GL Sciences, Tokyo, Japan). CD spectra (average of 16 spectra) were corrected for buffer signals.

### 3.6. 8-Anilino-1-Naphthalenesulfonic Acid (ANS) Fluorescence Assays

The ANS fluorescence was measured using Infinite M200 microplate reader (TECAN, Männedorf, Switzerland). ANS (FUJIFILM Wako Pure Chemical Corporation, Osaka, Japan, excitation wavelength of 371 nm and emission wavelength of 482 nm) was dissolved in buffer (50 mM Tris-HCl (pH 7.8 at 25 °C), 10 mM Mg(CH_3_COO)_2_, 20 mM KCl, 2 mM DTT). ANS was diluted to a final concentration of 5 μM. The buffer sample was used to determine a baseline measurement. The samples were placed in a 96-well microplate (polystyrene plate, black with transparent bottom; Greiner Bio-One, Kremsmünster, Austria) and inserted in the microplate reader, which scanned in 5 nm steps. The excitation was at 371 nm and the detector wavelength range was 400–600 nm.

### 3.7. Gel-Filtration Chromatography Analysis

Quaternary structures of Hsp60 WT and Hsp60 GW were investigated by gel-filtration chromatography with 50 mM Tris-HCl (pH 7.8 at 25 °C), 2 mM DTT, 2 mM EDTA. The samples (0.5 mg each) were applied to a Superdex 200 Increase 10/300 GL column (GE Healthcare Life Sciences, Marlborough, MA, USA). The flow rate was 0.75 mL/min, and the elution was detected by UV absorbance at 280 nm. Gel Filtration Standard (Bio-Rad, Hercules, CA, USA) was used for calibration standard for molecular weight markers.

### 3.8. Immunoprecipitation

The binding of Hsp60 WT/GW/AD(Cys) and α-synuclein was detected by immunoprecipitation combined with Western blot analysis. Hsp60 WT/GW/AD(Cys) and α-synuclein were agitated for 1 h before immunoprecipitation, and the mixture was used as the sample. The SureBeads™ Protein A Magnetic Beads (Bio-Rad, Hercules, CA, USA) were washed with PBS-T (Phosphate-Buffered Saline (Thermo Fisher Scientific, Waltham, MA, USA) + 0.1% Tween) three times, followed by addition of 200 μL PBS-T and 2 μL antibody (anti-Hsp60 (Mab11-13, mouse, abcam, Cambridge, UK for Hsp60 WT/GW detection), anti-6×His Tag (HIS.H8, mouse, Thermo Fisher Scientific, Waltham, MA, USA for AD(Cys) detection), or anti-α-synuclein (MJFR1, rabbit, abcam, Cambridge, UK)). After rotation for 10 min at 4 °C, the beads were magnetically retained, and the supernatant was discarded. The samples were added to the beads with bound antibodies and incubated with rotation for 4 h at 4 °C. The beads were again magnetically retained, and the supernatant was discarded. Then the beads were washed with PBS-T three times. Proteins were released from the beads by adding 2×SDS loading buffer and boiling for 3 min.

### 3.9. Western Blot Analysis

Proteins were separated by SDS-PAGE (15% gel) and transferred to PVDF membranes. Membranes were blocked with 5% skim milk in TBS-T (20 mM Tris-HCl (pH 8.0), 100 mM NaCl, 0.1% Tween 20) for 1 h at room temperature. The membrane was incubated at 4 °C overnight with appropriate primary antibodies: anti-Hsp60 (Mab11-13 (1/1000 dilution), mouse, abcam, Cambridge, UK for Hsp60 WT/GW detection), anti-6×His Tag (HIS.H8 (1/1000 dilution), mouse, Thermo Fisher Scientific, Waltham, MA, USA for AD(Cys) detection), anti-α-synuclein (MJFR1 (1/1000 dilution), rabbit, abcam, Cambridge, UK), or anti-α-Tubulin (DM1A (1/1000 dilution), mouse, Sigma-Aldrich, St. Louis, MO, USA). After washing with TBS-T, the membrane was incubated at room temperature with HRP-linked secondary antibodies: anti-rabbit IgG or anti-mouse IgG (1/10000 dilution, GE Healthcare Life Sciences, Marlborough, MA, USA) for 1 h. For detection, the membrane was incubated in Amersham ECL Prime Western Blotting Detection Reagent (GE Healthcare Life Sciences, Marlborough, MA, USA). Bands were photographed by ImageQuant LAS 4000mini (GE Healthcare Life Sciences, Marlborough, MA, USA). The image analysis software ImageQuant TL version 7.0 (GE Healthcare Life Sciences, Marlborough, MA, USA) was used for semi-quantitative analysis.

### 3.10. Quartz Crystal Microbalance (QCM) Assay

QCM (Affinix QN μ, ULVAC, Kanagawa, Japan) was used to directly detect interactions between α-synuclein and Hsp60 variants. To initialize the sensor cell, 1% sodium dodecyl sulfate and piranha solution (H_2_SO_4_:H_2_O_2_ = 3:1) were added on the gold surface. The interactions between Hsp60 WT or Hsp60 GW and His-α-synuclein were detected using an NTA/Ni_2_^+^ approach. Ni-NTA SAM (self-assembled monolayer) was formed by 0.5 mM Dithiobis (C_2_-NTA) and Ni buffer (10 mM NiSO_4_, 20 mM HEPES (pH 7.5), 150 mM NaCl, 50 mM EDTA). After equilibration of the sensor cell with a buffer (25 mM Tris buffer (pH 7.7 at 25 °C), 150 mM NaCl), His-α-synuclein was added. Then the sensor cell was cleared out for removal of unbound His-α-synuclein and subsequently fresh buffer was added. After frequency stabilization, Hsp60 WT or Hsp60 GW was added and measured. Before measuring the next sample, the sensor cell was filled with imidazole buffer (0.4 M imidazole, 20 mM HEPES (pH 7.5), 150 mM NaCl). Finally, final concentrations of 2.5, 5, 10, 15, and 20 nM Hsp60 were separately loaded into the sensor cell to monitor the frequency modulation.

Amine coupling method was employed for measuring the interaction between AD(Cys) and α-synuclein. After initializing the sensor cell, carboxylic acid-SAM formation reagent was added, then *N*-hydroxysuccinimide (NHS)/1-ethyl-3-(3-dimethylaminopropyl) carbodiimide (WSC) solution (NHS:WSC = 1:1) was added as an activation of the sensor surface. Then, 1 μM α-synuclein solution was applied and immobilized on the gold surface. Ethanolamine-solution was used to block residual activated esters. After frequency stabilization of the sensor cell with citrate buffer (pH 4.0), AD(Cys) was added and measured. Before measuring the next sample, 3 M Gdn-HCl was added into the sensor cell to remove AD(Cys). Frequency changes were measured for 2.5, 5, 10, 15, and 20 nM AD(Cys). AQUA analysis software version 2.0 (ULVAC, Kanagawa, Japan) was employed to calculate the K_d_, k_on_, and k_off_ values.

### 3.11. Cells

#### 3.11.1. Cell Cultures

Neuro2a (N2a) cells were obtained from Public Health England and were grown in Minimum Essential Medium (MEM, Thermo Fisher Scientific, Waltham, MA, USA) supplemented with 10% fetal bovine serum (FBS, Biological Industries, Kibbutz Beit-Haemek, Israel), MEM Non-Essential Amino Acid Solution (FUJIFILM Wako Pure Chemical Corporation, Osaka, Japan), 100 μM Sodium Pyruvate Solution (FUJIFILM Wako Pure Chemical Corporation, Osaka, Japan), and 100 U/mL Penicillin-Streptomycin (Thermo Fisher Scientific, Waltham, MA, USA). Cells were maintained at 37 °C in a humidified atmosphere with 5% CO_2_ condition.

#### 3.11.2. Construction of pCAG-GFP-Neo Gene

pCAG-GFP was a gift from Connie Cepko (Addgene, Watertown, MA, USA plasmid #11150; http://n2t.net/addgene:11150 (accessed on 18 December 2019); RRID:Addgene_11150) [54]. IRES2-AcGFP1-Nuc which included a neomycin/kanamycin resistance gene, was purchased from Takara Bio, Shiga, Japan. The PCR primers to amplify the templates are as follows: 5′ primer: 5′-CAT GCA TGT CGA CAT TGA TTA TTG ACT AGT TA-3′ and 3′ primer: 5′-GCC TCA GAG TGA GCG CAA CGC AAT TAA TGT-3′ for pCAG-GFP, 5′ primer: 5′-CGC TCA CTC TGA GGC GGA AAG AAC CAG CTG TG-3′ and 3′ primer: 5′-ATG TCG ACA TGC ATG GCG GTA ATA CGG TTA TCC A-3′ for pIRES2-AcGFP1-Nuc. The PCR amplified fragment of pCAG-GFP and pIRES2-AcGFP1-Nuc (containing neomycin/kanamycin resistance gene (Neo)) were fused by the In-Fusion method to generate pCAG-GFP-Neo plasmid. Finally, Xho I digestion site was introduced into pCAG-GFP-Neo plasmid between Rabbit Globin polyA and SV40 promoter by the overlap extension PCR method. The primers used were 5′-CAA CAC TCG AGC CGG AAG CAT AAA GTG T-3′ (forward) and 5′-CCG GCT CGA GTG TTG TGT GGA ATT GTG A-3′ (reverse).

#### 3.11.3. Generation of GFP-Syn Stable Cell Lines (GFP-Syn N2a Cells)

The pCAG-GFP-Neo plasmid was amplified using PCR. The primers were 5′-AGC GGC CGC ACT CCT CAG GTG CAG-3′ (forward) and 5′-CTT GTA CAG CTC GTC CAT GCC GAG AGT-3′ (reverse). The construction of pET23a-SNCA gene was referred to in our previous work [53]. The *SNCA*(Syn) gene was amplified using the following primers: 5′-GAC GAG CTG TAC AAG ATG GAT GTA TTC ATG AAA GGA CTT TC-3′ (forward) and 5′-AGG AGT GCG GCC GCT TTA GGC TTC AGG TTC GTA GTC TTG A-3′ (reverse). The PCR-amplified Syn gene was inserted into PCR-amplified pCAG-GFP-Neo vector by the In-Fusion method. The pCAG-GFP-Syn-Neo plasmid was linearized by digestion with Xho I enzyme, and the liner plasmid was introduced into N2a cells by electroporation (NEPA21 Super Electroporator, Nepa Gene, Chiba, Japan). Three days after transfection, the medium was replaced with medium containing 500 μg/mL G418 (Geneticin). Forty-eight colonies with GFP fluorescence were selected by a fluorescence microscope (ZEISS Axiovert 200, Oberkochen, Germany) and subcultured in a 48-well plate with 10% FBS + MEM. After the cells adhered to the plate, the medium was changed to 10% FBS + MEM + 500 μg/mL G418 and cultured to 80% confluence. Eighteen wells with strong GFP fluorescence were selected and passaged with 10% FBS + MEM. After culturing for two days, the medium was changed to 10% FBS + MEM + 500 μg/mL G418 medium and cultured until 80% confluent. One well was selected from 18 wells on the same basis, transferred to a flask and cultured to 80% confluence. Subsequently, the cells were further subcultured and was designated as the stable cell lines expressing GFP-Syn fusion protein (GFP-Syn N2a cells). It was confirmed by using a Tali™ Image-Based Cytometer (Thermo Fisher Scientific, Waltham, MA, USA) that 99% of cells had GFP fluorescence.

### 3.12. Introduction of Hsp60AD(Cys) Protein into Cells

The protein introduction procedure was performed according to the protocol recommended by the manufacturer. Briefly, the GFP-Syn N2a cells were cultured in six-well plate with glass coverslips, cultured until 80% confluent. A total of 30 μg of Hsp60AD(Cys) proteins were mixed with 3.0 μg of the “Prote-in” Transfection Reagent (Hygieia Bioscience, Osaka, Japan, Product Codes: 160-0526-0 and 160-0518-0). The mixture was incubated for 1 h at room temperature. Then it was added to each well, and incubated for 3 h at 37 °C. Aggregation of α-synuclein in GFP-Syn N2a cells were induced by exposure of 10 μM 6-hydroxydopamine (6-OHDA) for 24 h at 37 °C.

### 3.13. Measurement of Cell Death

The cell death measurement with EthD-1 staining was performed on a Tali™ Image-Based Cytometer (Thermo Fisher Scientific, Waltham, MA, USA). Three measurements were taken on different lots of cell culture samples. The cells were incubated with 400 nM EthD-1 (LIVE/DEAD™ Viability/Cytotoxicity Kit for mammalian cells, Thermo Fisher Scientific, Waltham, MA, USA) for 30 min in the dark. Then, 25 μL of cell suspension were injected into the measurement slide (Tali™ Cellular Analysis Slides, Thermo Fisher Scientific, Waltham, MA, USA). The measurement sensitivity and circularity settings were six and eight, respectively. The live control cells were treated in the same way except that neither AD(Cys) nor 6-OHDA was added. The threshold of EthD-1 fluorescence was set based on the peak value obtained from the live cell control. Cells that detected an EthD-1 fluorescence signal exceeding the threshold were defined as dead cells, and the dead cell rate was measured.

### 3.14. Immunofluorescence Experiments

Cells were fixed in 4% paraformaldehyde after nuclear staining using Hoechst 33342 (Dojindo Laboratories, Kumamoto, Japan). Cells were next permeabilized and blocked in 0.2% Triton X-100, 1% bovine serum albumin in PBS and then incubated with primary antibodies overnight at 4 °C. After washing with PBS, anti-mouse IgG specific secondary antibody (Alexa Fluor 647, abcam, Cambridge, UK) was added for 1 h at room temperature. The primary antibody used was specific against 6×Histidine tag (1:1000, HIS.H8, Thermo Fisher Scientific, Waltham, MA, USA). The secondary antibody used was anti-mouse IgG (1:1000, Alexa Fluor 647, abcam, Cambridge, UK). A drop of SlowFade Diamond Antifade Mountant (Thermo Fisher Scientific, Waltham, MA, USA) was added to the coverslip for antifade protection. Fluorescent staining was visualized using a confocal laser microscope (FLUOVIEW FV10i, Olympus, Tokyo, Japan).

## 4. Conclusions

In this study we demonstrated the suppression effect of α-synuclein amyloid formation by human Hsp60 mutant for the first time. Differences in the suppression effect according to the orientation of Hsp60 apical domain was also confirmed. Furthermore, our study is the first to characterize Hsp60 AD as a mini-chaperone. Our results could support further research on the use of Hsp60-based mini-chaperone as potential therapeutic agents for disease, such as PD. The investigation of the intracellular function of Hsp60 AD has only just begun. It is necessary to analyze various verifications in the future to explore the possibility of Hsp60 AD as a mini-chaperone.

## Figures and Tables

**Table 1:** The values K_d_, k_on_, and k_off_, estimated from linear regression model of the data shown in Figure 3b.

Sample	K_d_ (nM)	k_on_ (M^−1^s^−1^)	k_off_ (s^−1^)
Hsp60 WT	4.29	2.30 × 10^5^	9.89 × 10^−4^
Hsp60 GW	0.963	3.64 × 10^5^	3.51 × 10^−4^

**Table 2:** The values of K_d_, k_on_, and k_off_, estimated from linear regression of the data in Figure 5c.

Sample	K_d_ (nM)	k_on_ (M^−1^s^−1^)	k_off_ (s^−^^1^)
Hsp60 AD(Cys)	5.04	5.85 × 10^5^	2.95 × 10^−^^3^

## Supplementary Materials

Supplementary materials can be found at https://www.mdpi.com/1422-0067/21/1/47/s1.

## Abbreviations

AD	Apical domain
ANS	Anilino-1-naphthalenesulfonic acid
CD	Circular dichroism
C_2_-NTA	3,3′-Dithiobis[*N*-(5-amino-5-carboxypentyl) propionamide-*N*,*N*’-diacetic acid] dihydrochloride
EthD-1	Ethidium Homodimer-1
FBS	Fetal bovine serum
GFP-Syn	Green fluorescent protein-tagged human α-synuclein
GW	Hsp60 G190W mutant
IPTG	Isopropyl β-D-1-thiogalactopyranoside
MEM	Minimum Essential Medium
N2a	Neuro2a
Neo	Neomycin/kanamycin resistance gene
NHS	N-hydroxysuccinimide
6-OHDA	6-hydroxydopamine
PBS	Phosphate-Buffered Saline
PCR	Polymerase chain reaction
PD	Parkinson’s disease
PDB	Protein Data Bank
PMSF	Phenylmethylsulfonyl fluoride
polyQ	Polyglutamine
QCM	Quartz crystal microbalance
RFU	Relative Fluorescence Unit
SAM	Self-assembled monolayer
SEM	Standard error of the mean
sHsp	Small heat shock protein
TEM	Transmission Electron Microscopy
ThT	Thioflavin T
WSC	1-ethyl-3-(3-dimethylaminopropyl) carbodiimide
WT	Hsp60 wild type
