# Supplementary material for: Human Molecular Chaperone Hsp60 and Its Apical Domain Suppress Amyloid Fibril Formation of α-Synuclein
Source: Int J Mol Sci. 2019 Dec 19;21(1):47. doi: 10.3390/ijms21010047 (PMC6982183; doi:10.3390/ijms21010047)
Supplement: Supplementary file 1 [file ijms-21-00047-s001.pdf]

Supplementary Materials

Figure S1

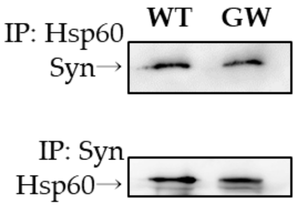

Figure S1. Demonstration of specific binding between Hsp60 and α-synuclein using immunoprecipitation and Western blot analysis. A mixture of Hsp60 and α-synuclein was subjected to immunoprecipitation with anti-Hsp60 antibody (upper panel) or anti-α-synuclein antibody (lower panel) followed by Western blot analysis using anti-α-synuclein antibody (upper panel) or anti-Hsp60 antibody (lower panel) to detect the Hsp60 - α-synuclein interaction (upper panel) or the α-synuclein - Hsp60 interaction (lower panel).

Figure S2

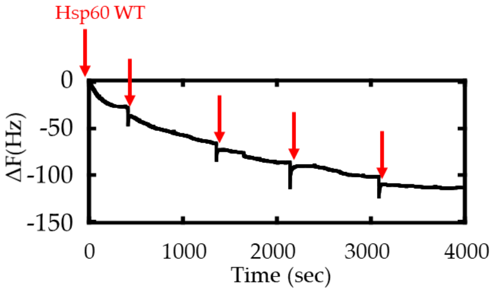

	K_d (M)
Hsp60WT	8.3×10^{-8}

Figure S2. Determination of the dissociation constant (K_d) between Hsp60 and Hsp10 in the presence of 1 mM ATP. The sensorgram (ΔF against time) during sequential titration by Hsp60 WT aliquots (1 μM, 5 μL) to achieve saturation of bound Hsp10 is shown. Hsp10 was immobilized on the sensor by amine coupling and the K_d of Hsp60 WT/Hsp10 in the presence of 1 mM ATP was measured. The red arrow indicates the time when Hsp60WT was added. The K_d value (8.3×10^{-8} M) was obtained with the affinity analysis software AQUA provided with the QCM instrument. Since Ishida et al. reported that the K_d value of Hsp60/Hsp10 measured by fluorescence cross-correlation spectroscopy (FCCS) analysis was 8.7×10^{-8} M (Int. J. Mol. Sci., 2018, 19, 489), the K_d value measured by QCM agrees well with the K_d value measured by FCCS.

Figure S3

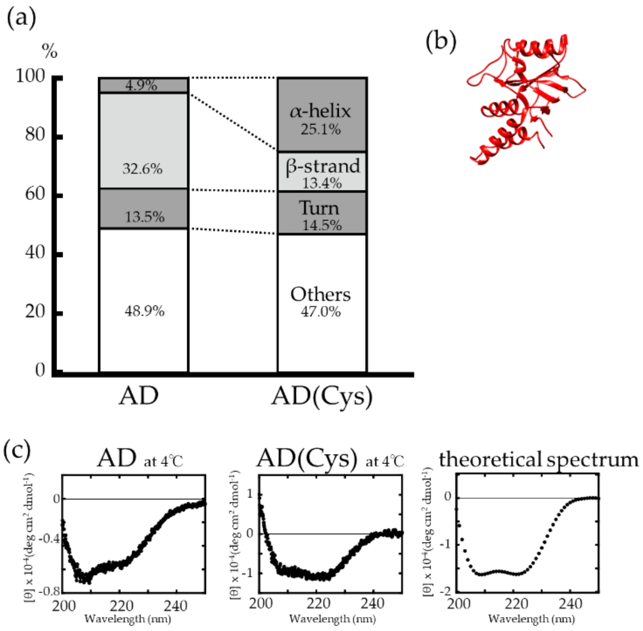

Figure S3. (a) CD spectra deconvolution by BeStSel software for AD and AD(Cys) at 4 °C (b) Three-dimensional structure of Hsp60AD revealed by crystal structure analysis (PDB ID: 4PJ1, resolution: 3.15 Å) (c) left and middle: CD spectrum at 4 °C of AD and AD(Cys). right: The theoretical CD spectrum drawn based on the crystal structure of AD using the CD spectrum simulation program (J Chem Educ, 2011, 88, 1268-1273).

The secondary structure components of AD and AD(Cys) were analyzed by the protein secondary structure prediction web server, Beta Structure Selection (BeStSel, Nucleic Acids Res., 2018, 46, W315-W322). Figure S3(a) shows the composition ratio of secondary structure contents expected from each CD spectrum at 4 °C. Comparing the composition ratio of AD and AD(Cys), the ratio of α-helix and β-strand is significantly different. Figure S3(b) shows that the three-dimensional structure of Hsp60AD revealed by crystal structure analysis (PDB ID: 4PJ1, resolution: 3.15 Å), and indicates that AD contains much α-helical content. Figure S3(c) shows CD spectrum at 4 °C of AD and AD(Cys), and the theoretical CD spectrum drawn based on the crystal structure of AD. We drew the theoretical CD spectrum of AD using the CD spectrum simulation program (J. Chem. Educ., 2011, 88, 1268-1273). The CD spectrum of AD(Cys) is close to the theoretical CD spectrum of AD. The slight difference in CD spectra between AD(Cys) and the theoretical spectrum may be attributed to the disulfide bond in AD(Cys). In this context, the structure of AD seems to be partially unfolded even at 4 °C.

Figure S4

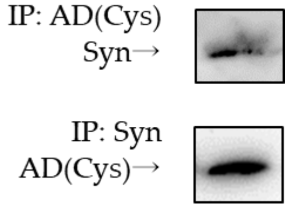

Figure S4. Demonstration of specific binding between AD(Cys) and α-synuclein using immunoprecipitation and Western blot analysis. The mixture of AD(Cys)(-His) and α-synuclein was subjected to immunoprecipitation with anti-6 × His Tag antibody (upper panel) or anti-α-synuclein antibody (lower panel) followed by Western blot analysis using anti-α-synuclein antibody (upper panel) or anti-6xHis Tag antibody (lower panel) to detect the AD(Cys) - α-synuclein interaction (upper panel) or the α-synuclein - AD(Cys) interaction (lower panel).

Figure S5

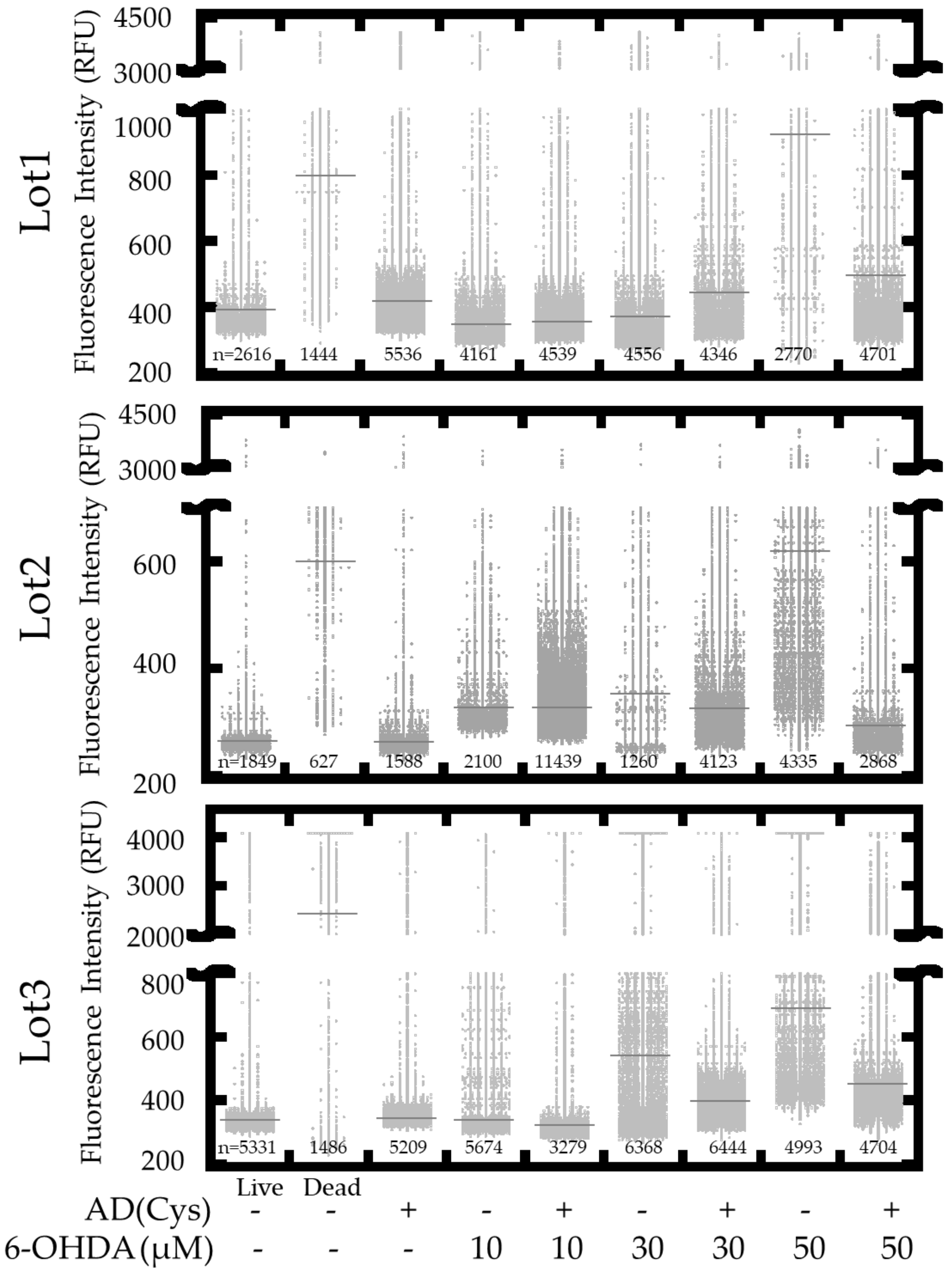

Figure S5. Comparison of fluorescence intensity of ethidium homodimer 1 with and without AD(Cys) using three different lots of cells. *n* indicates the number of cells measured. The line superimposed on the dot plot shows the median value. “Live” indicates the live cell controls that were treated in the same way except that neither AD(Cys) nor 6-OHDA was used. “Dead” indicates the dead cell controls that were treated with 70% ethanol for 30 min.

Figure S6

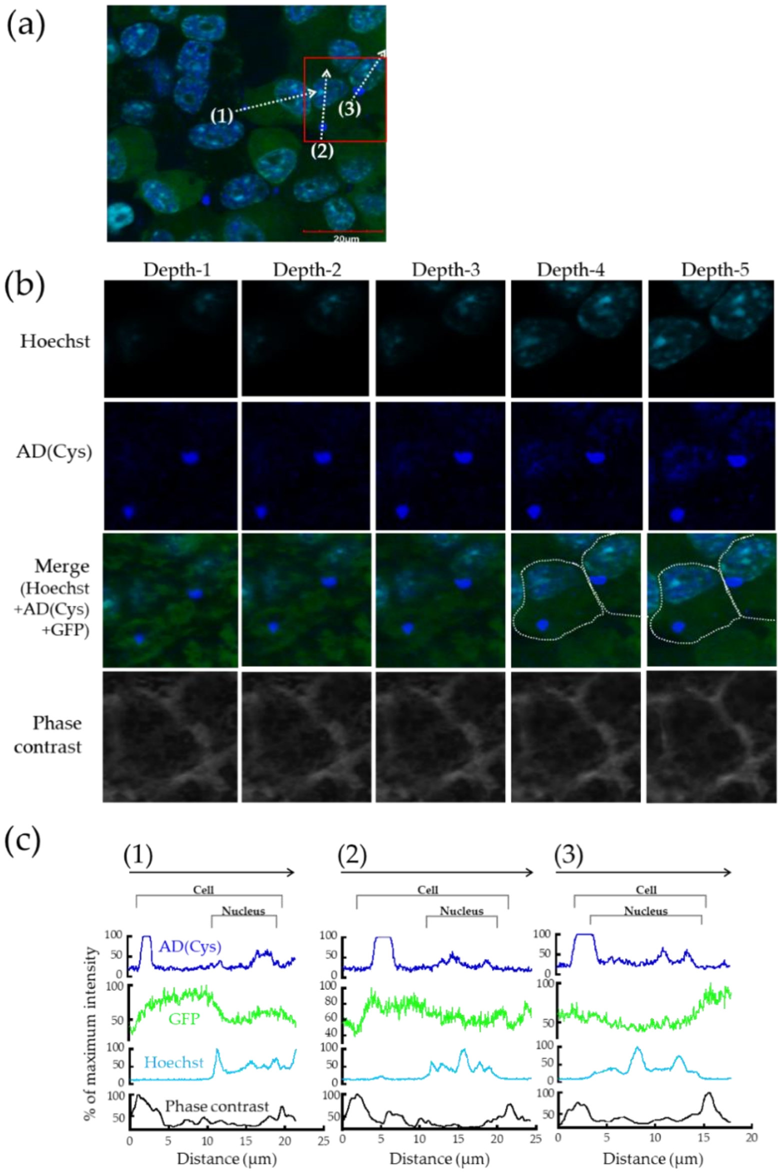

Figure S6. Image analysis of AD(Cys) introduced cells. (a) AD(Cys) was introduced into cells and oxidative stress was then applied by addition of 10 μM 6-OHDA. The confocal laser microscope image displays intrinsic fluorescence of GFP-*α*-synuclein (green), immunofluorescence of AD(Cys) (blue) and nuclear labelling using Hoechst 33342 (cyan). The white arrows (1), (2) and (3) denote the cross-sections sampled that transect the nucleus which are illustrated in (c). Scale bar in (a): 20 μm. (b) Serial Z-stack images of the red boxed region in (a). (Z-stack: 1 μm/slice) (c) Cross-section fluorescence intensities of the three locations denoted by the arrows in (a). Values are normalized to the maximum intensity of the respective signals.

Figure S7

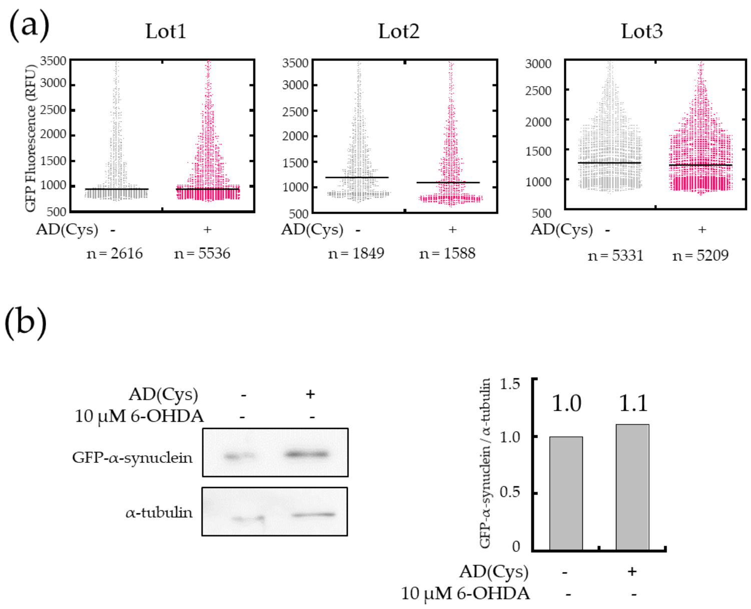

Figure S7. Comparison of GFP fluorescence intensity and GFP-α-synuclein expression in cells with and without introduced AD(Cys). (a) Comparison of fluorescence intensity of GFP-α-synuclein of three different cell cultures. n indicates the number of cells measured. The horizontal line within each plot denotes the median value. (b) Western blot experiment to examine changes in expression of GFP-α-synuclein by introducing AD(Cys). The bar graph on the right shows the ratio of GFP-α-synuclein to α-tubulin quantified with a digital image analyzer.
